# Angiotensin-Converting Enzyme Inhibitors and Angiotensin Receptor Blockers in Acute Coronary Syndrome: Implications for Platelet Reactivity?

**DOI:** 10.1007/s10557-020-07128-0

**Published:** 2020-12-18

**Authors:** Maximilian Tscharre, Patricia P. Wadowski, Constantin Weikert, Joseph Pultar, Beate Eichelberger, Simon Panzer, Thomas Gremmel

**Affiliations:** 1 Department of Internal Medicine, Cardiology and Nephrology, Landesklinikum Wiener Neustadt, Wiener Neustadt, Austria; 2 Department of Internal Medicine II, Medical University of Vienna, Waehringer Guertel 18-20, 1090 Vienna, Austria; 3 Department of Blood Group Serology and Transfusion Medicine, Medical University of Vienna, Vienna, Austria; 4 Department of Internal Medicine I, Cardiology and Intensive Care Medicine, Landesklinikum Mistelbach-Gänserndorf, Mistelbach, Austria

**Keywords:** Angiotensin-converting enzyme inhibitors, Angiotensin receptor blockers, Acute coronary syndromes, Multiple electrode aggregometry, Platelet reactivity

## Abstract

**Background:**

In patients with acute coronary syndrome (ACS), angiotensin-converting enzyme (ACE) inhibitors are preferred over angiotensin receptor blockers (ARBs). However, in a recent pilot study, treatment with ACE inhibitors was associated with increased platelet reactivity compared to ARBs. Therefore, we sought to investigate the impact of renin-angiotensin-aldosterone system (RAAS) blockade with ACE inhibitors and ARBs on platelet aggregation in patients with ACS undergoing percutaneous coronary intervention.

**Methods:**

On-treatment residual platelet reactivity in response to arachidonic acid (AA), adenosine diphosphate (ADP), SFLLRN, AYPGKF, and collagen was assessed by multiple electrode aggregometry (MEA) in 197 ACS patients on dual antiplatelet therapy (DAPT) with aspirin and either prasugrel or ticagrelor.

**Results:**

One hundred sixty-five (83.7%) patients were treated with ACE inhibitors, 32 (16.3%) with ARBs. On-treatment residual AA- and ADP-inducible platelet reactivity was significantly higher in patients with ACE inhibitors (both *p* < 0.05). Likewise, SFLLRN was significantly higher in patients with ACE inhibitors (*p* = 0.036) and there was a trend for higher AYPGKF- and collagen-inducible platelet reactivity (*p* = 0.053 and *p* = 0.082). The incidence of high on-treatment residual platelet reactivity AA was significantly higher in patients with ACE inhibitors (52 [31.5%] vs. 3 [9.4%] patients; *p* = 0.019).

**Conclusion:**

ACE inhibitors are associated with increased on-treatment residual platelet reactivity in ACS patients with potent DAPT. Further clinical trials are needed to elucidate the role of RAAS blockade with ACE inhibitors and ARBs in ACS patients treated according to current standards.

## Introduction

Dual antiplatelet therapy (DAPT) with aspirin and a P2Y12 inhibitor is the antithrombotic standard regimen for patients presenting with acute coronary syndromes (ACS) undergoing percutaneous coronary intervention (PCI) [1–3]. Prasugrel and ticagrelor are newer adenosine diphosphate (ADP) P2Y12 receptor antagonists that have been shown to be superior compared to clopidogrel in reducing adverse cardiovascular outcomes in ACS due to faster, stronger, and more consistent inhibition of ADP-induced platelet activation [4, 5]. However, atherothrombotic events still impair the prognosis of many patients with ACS despite state-of-the-art antiplatelet therapy [6]. While high on-treatment residual platelet reactivity (HRPR) to ADP is a rare phenomenon in patients treated with prasugrel or ticagrelor [7], subsequent ischemic events in patients receiving the novel P2Y12 antagonists may in part be attributable to platelet activation via other, non-inhibited pathways [8–10]. In addition, previous studies have identified several concomitant pharmacological therapies potentially attenuating the antiplatelet effects of clopidogrel [11–14].

According to current guidelines, renin-angiotensin-aldosterone system (RAAS) blockade with an angiotensin-converting enzyme (ACE) inhibitor or an angiotensin receptor blocker (ARB) should be considered in all patients presenting with ST-elevation myocardial infarction (STEMI) and is recommended for all ACS patients suffering from either arterial hypertension, chronic kidney disease, diabetes, or reduced left-ventricular ejection fraction [2, 3]. Moreover, ARBs should only prescribe in case of intolerance against ACE inhibitors [2, 3].

However, in a recent pilot-analysis by Helten et al. including patients with an indication for RAAS blockade (arterial hypertension or heart failure), ACE inhibitors were associated with increased platelet surface expression of protease-activated receptor-1 (PAR-1) and enhanced SFLLRN (=thrombin receptor activating peptide [TRAP])-inducible platelet reactivity compared to ARBs [15].

Considering these recent data, we sought to assess the impact of RAAS blockade with ACE inhibitors or ARBs on platelet aggregation in ACS patients receiving either prasugrel or ticagrelor following acute PCI.

## Methods

### Patient Population

The study population consisted of 197 ACS patients on daily aspirin (100 mg/day), and either prasugrel (10 mg/day) or ticagrelor (180 mg/day) therapy. All study patients were of Caucasian ethnicity. Blood sampling was performed 72 h after acute PCI with stent implantation. Due to the short half-life of unfractionated heparin, all patients were free of heparin from PCI at the time of blood sampling [16].

Exclusion criteria were a known P2Y12 inhibitor or aspirin intolerance (manifested as allergic reactions or gastrointestinal bleeding); a therapy with vitamin K antagonists (phenprocoumon, acenocoumarol, warfarin), rivaroxaban, apixaban, dabigatran, or edoxaban; treatment with nonsteroidal anti-inflammatory drugs, ticlopidine, or dipyridamole; known bleeding disorders; severe hepatic failure; known qualitative defects in platelet function; heparin-induced thrombocytopenia; malignant myeloproliferative disorders; a platelet count < 100,000 or > 450,000/μL; a hematocrit < 30%; and a major surgical procedure within 1 week before enrollment.

### Blood Sampling

Blood was drawn by aseptic venipuncture from an antecubital vein using a butterfly needle (21 gauge, 0.8 × 19 mm; Greiner Bio-One, Kremsmünster, Austria) 72 h after PCI, as described previously [9]. To avoid procedural deviations, blood sampling was performed by the same physician applying a light tourniquet, which was immediately released, and the samples were mixed by gently inverting the tubes. The initial 3 mL of blood was discarded to reduce periprocedural platelet activation. Afterwards, blood was drawn into hirudin-coated tubes (Roche Diagnostics, Mannheim, Germany) for multiple electrode aggregometry (MEA).

### Multiple Electrode Aggregometry

Whole blood impedance aggregometry was performed using the Multiplate analyzer (Roche Diagnostics) as previously described [10]. In brief, hirudin-anticoagulated whole blood was diluted 1:2 with 0.9% NaCl solution and stirred in the test cuvettes for 3 min at 37 °C. Thereafter, arachidonic acid (AA; 0.5 mM), ADP (6.4 μM), collagen (2.7 μg/mL), SFLLRN (PAR-1 agonist, 32 μM), or AYPGKF (PAR-4 agonist, 645 μM, all from Roche Diagnostics) was added and aggregation was recorded for 6 min. Titration experiments were carried out, increasing the dosages of SFLLRN and AYPGKF, respectively, until both agonists induced platelet aggregation > 60 aggregation units (AU) by MEA, but less than maximal response in healthy Caucasian individuals (*n* = 30). The determined dosages corresponded to the concentrations recommended by the manufacturer. The interaction of activated platelets with the electrode led to an increase of impedance, which was detected for each sensor unit separately and transformed to aggregation units (AU) that were plotted against time. The AU at 6 min were used for calculations. One AU corresponds to 10 AU*min (area under the curve of AU).

### Statistical Analysis

All continuous variables are expressed as median (interquartile range [IQR]). Categorical variables are given as number (%). Continuous variables were compared by Mann-Whitney *U* test for independent samples. χ^2^ test or Fisher’s exact test was performed for comparison of categorical variables, as appropriate. Multivariable linear regression analyses using a backward elimination algorithm with a *p* value ≤ 0.1 for removal were used to adjust for patient characteristics. Adjustment was performed for the following variables: age, sex, body mass index, hypertension, hyperlipidemia, diabetes, active smoking, peripheral artery disease, prior myocardial infarction, prior stroke or transient ischemic attack, number of affected coronary vessels, type of P2Y12 inhibitor, use of statins, beta blockers, or proton pump inhibitors. All statistical tests were 2-tailed and a *p* value < 0.05 was considered significant. All statistical analyses and figures were generated with Statistical Package for Social Sciences (IBM SPSS version 24, Armonk, NY, USA) and R 3.6.3.

## Results

In total, 197 patients were eligible for analysis. Of all patients, 165 (83.7%) patients were treated with an ACE inhibitor, 32 (16.3%) with an ARB. Median age was 57 (IQR 49–66) years, and 36 (18.3%) were female. Prasugrel was prescribed to 113 (57.4%), and ticagrelor to 84 (42.6%) patients. Clinical, laboratory, and procedural characteristics of the overall study population and stratified for patients with ARBs and ACE inhibitors are presented in Table 1.

**Table 1:** Baseline characteristics

	All patients	ARB	ACE-I	*p* value
	*N* = 197	*N* = 32	*N* = 165	
Age, years	57 (49–66)	62 (54–72)	56 (48–64)	0.003
Sex, No. (%):				0.409
Female patients	36 (18.3%)	8 (25.0%)	28 (17.0%)	
Male patients	161 (81.7%)	24 (75.0%)	137 (83.0%)	
Body mass index, kg/m^2^	27.8 (25.2–30.4)	29.4 (26.7–32.3)	27.6 (25.1–29.9)	0.035
Hemoglobin, g/dl	14.0 (13.0–14.7)	13.8 (12.5–14.6)	14.0 (13.1–14.8)	0.322
Leukocyte count, G/l	8.9 (7.4–10.4)	8.6 (7.5–9.9)	9.0 (7.3–10.6)	0.430
Creatinine, mg/dl	0.9 (0.8–1.1)	1.1 (0.9–1.4)	0.9 (0.8–1.0)	< 0.001
High sensitivity C-reactive protein, mg/l	1.17 (0.56–3.59)	1.37 (0.86–3.15)	1.12 (0.55–3.75)	0.556
proBNP, pg/ml	658 (255–1285)	675 (267–1357)	656 (256–1269)	0.739
Diabetes mellitus, No. (%)	50 (25.8%)	9 (29.0%)	41 (25.2%)	0.819
Arterial hypertension, No. (%)	135 (69.2%)	29 (90.6%)	110 (66.7%)	0.008
Hyperlipidemia, No. (%)	148 (76.7%)	24 (77.4%)	124 (76.5%)	1.000
Prior myocardial infarction, No. (%)	32 (16.4%)	10 (31.2%)	22 (13.5%)	0.027
Prior stroke or TIA, No. (%)	6 (3.1%)	2 (6.2%)	4 (2.5%)	0.595
Peripheral artery disease, No. (%)	13 (6.8%)	3 (9.7%)	10 (6.3%)	0.693
Use of DES, No. (%):	193 (98.5%)	31 (96.9%)	162 (98.8%)	0.406
Affected coronary vessels, No. (%)	2 (1–2)	2 (2–2)	2 (1–2)	0.005
Prasugrel, No. (%)	113 (57.4%)	15 (46.9%)	98 (59.4%)	0.265
Ticagrelor, No. (%)	84 (42.6%)	17 (53.1%)	67 (40.6%)	0.265
Beta blocker, No. (%)	191 (97.0%)	30 (93.8%)	161 (97.6%)	0.261
Statin, No. (%)	195 (99.0%)	32 (100%)	163 (98.8%)	1.000

Data are presented as median (IQR) or *n* (%). *ACE*, angiotensin-converting enzyme; *ARB*, angiotensin receptor blocker; *BNP*, brain natriuretic peptide; *DES*, drug-eluting stents; *TIA*, transient ischemic attack

**Fig. 1:**
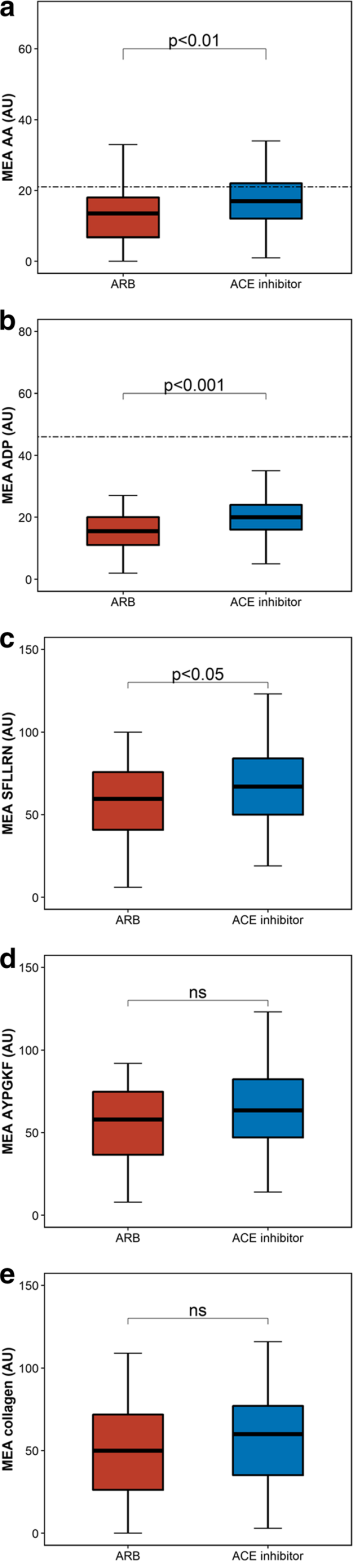
Aggregation units (AU) by multiple electrode aggregometry (MEA) in response to AA (panel **a**), ADP (panel **b**), SFLLRN (panel **c**), AYPGKF (panel **d**), and collagen (panel **e**) stratified for patients with angiotensin-converting enzyme (ACE) inhibitors and angiotensin receptor blockers (ARBs). Cut-off values for high on-treatment residual platelet reactivity are indicated by the dashed lines. The boundaries of the box show the lower and upper quartile of data, and the line inside the box represents the median. Whiskers are drawn from the edge of the box to the highest and lowest values that are outside the box but within 1.5 times the box length

On-treatment residual AA- and ADP-inducible platelet reactivity was significantly higher in patients with ACE inhibitors as compared to patients with ARBs (AA: 17 AU [12–22] vs. 13 AU [7–18], *p* = 0.006; ADP: 20 AU (16–24) vs. 16 AU (11–20), *p* < 0.001; Table 2, Fig. 1). Likewise, SFLLRN-inducible platelet reactivity was significantly higher in patients with ACE inhibitors (67 AU [50–84] vs. 60 AU [41–76], *p* = 0.036; Table 2, Fig. 1), and there was a trend for higher AYPGKF- and collagen-inducible platelet reactivity (AYPGKF: 64 AU [47–82] vs. 58 AU [37–75], *p* = 0.053; collagen: 60 AU [35–77] vs. 50 AU [26–72], *p* = 0.082) in patients on ACE inhibitors. In multivariable linear regression analysis, ACE inhibitor therapy remained significantly associated with increased AA- and ADP-inducible platelet reactivity (both *p* < 0.05), whereas after adjustment, no association with SFLLRN-, AYPGKF-, and collagen-induced platelet reactivity was detectable (all *p* > 0.05).

**Table 2:** Agonist-inducible platelet reactivity stratified according to patients with ARBs and ACE inhibitors

	ARB	ACE-I	*p* value
	*N* = 32	*N* = 165	
MEA AA 5 mM, AU	13 (7–18)	17 (12–22)	0.006
MEA ADP 6.4 μM, AU	16 (11–20)	20 (16–24)	< 0.001
MEA COL, AU	50 (26–72)	60 (35–77)	0.082
MEA AYPGKF 645 μM, AU	58 (37–75)	64 (47–82)	0.053
MEA SFLLRN 32 μM, AU	60 (41–76)	67 (50–84)	0.036

Data are presented as median (IQR). *AA*, arachidonic-acid; *ACE*, angiotensin-converting enzyme; *ADP*, adenosine diphosphate; *ARB*, angiotensin receptor blocker; *MEA*, multiple electrode aggregometry

High on-treatment residual platelet reactivity (HRPR) to AA and ADP was defined according to previous studies showing an association between platelet aggregation by MEA and ischemic outcomes following PCI [17, 18]. The respective cut-off values were AU ≥ 21 and > 46 for MEA AA and MEA ADP, respectively. With use of these thresholds, the frequency of HRPR ADP was similar between patients with ACE inhibitors and ARBs (2 vs. 0 patients; *p* = 1.000), whereas the incidence of HRPR AA was significantly higher in patients on ACE inhibitors (52 [31.5%] vs. 3 [9.4%] patients; *p* = 0.019).

In prasugrel-treated patients, ADP-, AYPGKF-, and SFLLRN-inducible platelet reactivity was significantly higher in those receiving ACE inhibitors as compared to patients on ARBs (all *p* < 0.05; Table 3), and there was a trend towards higher AA- and collagen-inducible platelet reactivity (AA: *p* = 0.058; collagen: *p* = 0.054) in patients on ACE inhibitors. However, after adjustment, ACE inhibitors no longer were associated with agonist-inducible residual platelet reactivity in patients treated with prasugrel (all *p* > 0.05).

**Table 3:** Agonist-inducible platelet reactivity stratified according to patients with ARBs and ACE inhibitors and according to antiplatelet therapy

	Prasugrel			Ticagrelor		
	ARB	ACE-I	*p* value	ARB	ACE-I	*p* value
	*N* = 15	*N* = 98		*N* = 17	*N* = 67	
MEA AA 5 mM, AU	14 (8–18)	17 (13–22)	0.056	13 (7–16)	17 (12–21)	0.082
MEA ADP 6.4 μM, AU	17 (11–21)	20 (16–23)	0.047	15 (12–20)	22 (16–25)	0.003
MEA COL, AU	44 (19–55)	60 (34–77)	0.054	58. (34–74)	61 (37–78)	0.469
MEA AYPGKF 645 μM, AU	53 (30–75)	61 (47–84)	0.037	61 (40–75)	65 (47–79)	0.443
MEA SFLLRN 32 μM, AU	46 (33–78)	68 (53–85)	0.025	60 (51–67)	64 (48–78)	0.608

Data are presented as median (IQR). *AA*, arachidonic-acid; *ACE*, angiotensin-converting enzyme; *ADP*, adenosine diphosphate; *ARB*, angiotensin receptor blocker; *MEA*, multiple electrode aggregometry

In patients on ticagrelor, residual AA-inducible platelet reactivity was numerically but not statistically and ADP-inducible reactivity significantly higher in patients with ACE inhibitors. SFLLRN-, AYPGKF-, and collagen-inducible platelet reactivity was similar in patients with ACE inhibitors and ARBs (Table 3). In multivariable regression analysis, ACE inhibitors remained associated with ADP-inducible platelet reactivity (*p* = 0.002) but were not associated with AA-, SFLLRN-, AYPGKF-, and collagen-inducible residual platelet reactivity in patients on ticagrelor (all *p* > 0.05).

## Discussion

This is the first study investigating the association of ACE inhibitors and ARBs with on-treatment residual platelet reactivity in ACS patients on potent DAPT undergoing PCI. Our main finding was that agonist-inducible platelet reactivity as assessed by MEA was higher in patients receiving ACE inhibitors as compared to patients receiving ARBs. Moreover, there was an increased incidence of HRPR AA in patients with ACE inhibitors.

The current antithrombotic therapy regimen following ACS with PCI includes aspirin and a potent P2Y12 inhibitor, either prasugrel or ticagrelor [2, 3]. Aspirin irreversibly acetylates a serine residue of cyclooxygenase (COX) 1 and COX 2, and inhibits thromboxane A2 (TXA2) generation by suppressing the synthesis of prostaglandin G2 and H2 [19]. Prasugrel and ticagrelor bind P2Y12, a G protein–coupled receptor for ADP, which is responsible for sustained amplification and stabilization of platelet aggregation [20]. However, platelets can be activated by various pathways and agonists, and residual platelet reactivity resulting in subsequent atherothrombotic events still impair the prognosis in many patients suffering from cardiovascular disease [20].

The beneficial effects of ACE inhibitors on vasoconstriction, cell growth, sodium and water retention, and sympathetic activation result from the suppression of angiotensin II formation, and consequently the downstream inhibition of the angiotensin II type 1 (AT1) and type 2 (AT2) receptors [21]. In contrast, ARBs specifically target the AT1 receptor and additionally trigger vasodilatation and natriuresis by facilitating the stimulation of the AT2 receptor [21]. In ACS patients, ACE inhibitors were associated with a small but significant reduction in 30-day mortality [22]. Moreover, in patients with ACS and systolic dysfunction, treatment with ARBs was not associated with a benefit in patient outcomes compared to ACE inhibitors [23, 24]. Finally, there are no clinical trials investigating ARBs in ACS patients with preserved left-ventricular function. Therefore, according to current guidelines, ACE inhibitors are the first choice; only if not tolerated ARBs shall be prescribed [2, 3]. However, in a recent propensity-matched analysis and a recent meta-analysis, ARBs demonstrated similar efficacy and superior safety compared to ACE inhibitors [25, 26]. Moreover, in line with our findings, a recent pilot study including 34 patients with arterial hypertension or heart failure treatment with an ACE inhibitor (ramipril) demonstrated significantly increased SFLLRN-inducible platelet reactivity already 4 h after treatment initiation, which remained elevated during therapy [15]. These findings were the result of reduced thrombin formation in response to ACE inhibitor therapy, which in turn enhanced PAR-1 surface expression on platelets leading to increased platelet reactivity. Interestingly, after switching to an ARB (candesartan) SFLLRN-inducible platelet reactivity and thrombin receptor, expression decreased significantly [15]. Of note, not all patients were on antiplatelet therapy (65% patients with aspirin, 44% patients with P2Y12 inhibitor) [15]. However, we previously demonstrated that platelet activation via the PAR1- and PAR-4 pathways remains active in many patients on DAPT, even in patients receiving the more potent P2Y12 inhibitors prasugrel and ticagrelor [9, 10, 27]. In our cohort with all patients on potent DAPT, SFLLRN-inducible platelet aggregation was higher in patients receiving ACE inhibitors. This is of particular interest, as in a former study, PAR-1-mediated platelet activation was associated with adverse outcomes in patients with peripheral angioplasty and stent implantation [28]. Moreover, treatment with vorapaxar (a PAR-1 antagonist) on top of DAPT significantly reduced the composite endpoint of myocardial infarction, stroke, and cardiovascular death in ACS patients in the TRACER trial, but also significantly increased the risk of bleeding including intracranial hemorrhage [29].

In a randomized controlled study including aspirin-treated patients with coronary artery disease (CAD; defined as < 50% stenosis in coronary angiography) comparing the impact RAAS blockade on platelet aggregation, irbesartan (an ARB) significantly reduced TXA2-induced platelet aggregation, whereas enalapril (an ACE inhibitor) had no effect on TXA2-induced platelet aggregation assessed by turbidometry [30]. As urinary prostaglandin E2 levels were unaffected, the authors suspected a COX-2 independent mechanism by either direct inhibitory effects of irbesartan or its active metabolites at the TXA2-receptor [30]. Likewise, in our cohort, we detected higher on-treatment residual AA- and ADP-inducible platelet reactivity by MEA in patients with ACE inhibitor therapy. Moreover, patients with ACE inhibitors had a higher incidence of HRPR AA compared to patients with ARBs. This is of great interest, as residual AA- and ADP-inducible platelet reactivity has repeatedly been associated with adverse ischemic outcomes in patients undergoing PCI and stenting on DAPT [17, 31–33].

It is noteworthy that no widely accepted threshold to identify patients with HRPR AA has been defined to date and higher thresholds than those used in our manuscript have been reported in the literature (MEA AA > 30 AU or > 40 AU, respectively) [34, 35]. However, these cut-offs derive from small analyses comparing healthy controls with patients on aspirin monotherapy and stable cardiovascular disease, and might not apply to patients with a recent acute ischemic event. Moreover, these cut-offs have not been validated in a clinical outcome trial. We therefore defined HRPR AA according to a recent report by Mayer et al. including patients undergoing PCI on DAPT, in which MEA AA < 21 AU was associated with a higher risk for death or stent thrombosis during the first year after PCI [17].

The use of prasugrel and ticagrelor has been associated with low ADP-inducible platelet reactivity in recent studies by others and us [9, 36, 37]. In line with these previous studies, HRPR ADP was only seen in 2 patients in the current study. In this regard, however, it should be kept in mind that all thresholds for HRPR in response to ADP were defined in clopidogrel-treated patients and may not apply to patients receiving prasugrel or ticagrelor. Furthermore, more potent platelet inhibition by the modern P2Y12 inhibitors (prasugrel and ticagrelor) was superior to clopidogrel in reducing thrombotic events and cardiovascular death, but increased the risk of bleeding complications [4, 5]. One may therefore speculate that more intense platelet inhibition as seen in patients with ARBs might result in a further clinical benefit regarding the reduction of ischemic outcomes in patients on potent P2Y12 inhibitors, potentially at the cost of an increased risk of bleeding complications.

ACE inhibitors are currently preferred over ARBs in the setting of ACS. However, these data derive from randomized clinical trials, which do not apply current standards including treatment with primary PCI for the majority of patients and potent DAPT with prasugrel and ticagrelor. Our data indicate a benefit of ARBs over ACE inhibitors with respect to on-treatment residual platelet reactivity in ACS patients with potent DAPT. Further clinical trials are needed to elucidate the role of RAAS blockade with ACE inhibitors and ARBS in ACS patients treated according to modern standards.

## Limitations

Our data must be regarded as hypothesis-generating only and further investigations are needed, as this study cannot prove causality nor exclude other possible confounders. The present investigation should be interpreted with the following limitations in mind: Our study is not powered nor intended to provide clinical outcome data. Also, we measured platelet aggregation at only one time point using a single test system. Moreover, we did not perform in vivo analyses. However, MEA is a highly standardized platelet function test ensuring a good comparability of the obtained results with other laboratories, and platelet aggregation by MEA has repeatedly been linked to cardiovascular outcomes following PCI [17, 31].

## Conclusion

ACE inhibitors are associated with increased on-treatment residual platelet reactivity in ACS patients on potent DAPT. Further clinical trials are needed to elucidate the role of RAAS blockade with ACE inhibitors and ARBs in ACS patients treated according to modern standards.

## Data Availability

All participants were enrolled at the Department of Internal Medicine II of the Medical University of Vienna. The data underlying this article will be shared on reasonable request to the corresponding author.
